# Pseudorheumatoid nodules treated with abrocitinib

**DOI:** 10.1016/j.jdcr.2025.06.023

**Published:** 2025-06-24

**Authors:** Samantha Bizimungu, Meryem Safoine, Kevin Watters, Alexandre Lemieux

**Affiliations:** a Division of Dermatology, Department of Medicine, Centre hospitalier de l’Université de Montréal, Montreal, Quebec, Canada; b Department of Pathology, McGill University Health Center, Montreal, Quebec, Canada; c Division of Dermatology, Department of Medicine, Hôpital du Sacré-Cœur de Montréal, Montreal, Quebec, Canada

**Keywords:** abrocitinib, JAK inhibitor, pseudorheumatoid nodules, subcutaneous granuloma annulare

## Introduction

Pseudorheumatoid nodules are an uncommon clinicopathological entity considered to be a variant of subcutaneous granuloma annulare.^1^ They predominantly affect children, but adult cases have been reported. They present as painless, skin-colored subcutaneous nodules most commonly on the extremities. Adults are more likely to have multiple lesions in a juxta-articular distribution, mimicking rheumatoid nodules. Histopathology shows nodules of palisading granulomas surrounding degenerated collagen in the deep dermis and hypodermis, with scant mucin deposits. A clinical and a serologic evaluation should be performed to exclude rheumatic disease. Surgical excision is the most described treatment modality, although recurrence occurs in up to 38% of cases.^2^ Many nonsurgical treatments have been reported, including topical, intralesional, and oral steroids; chloroquine; dapsone; and pentoxifylline, with limited efficacy. We present a case of long-lasting pseudorheumatoid nodules successfully treated with abrocitinib.

## Case report

**Fig 1:**
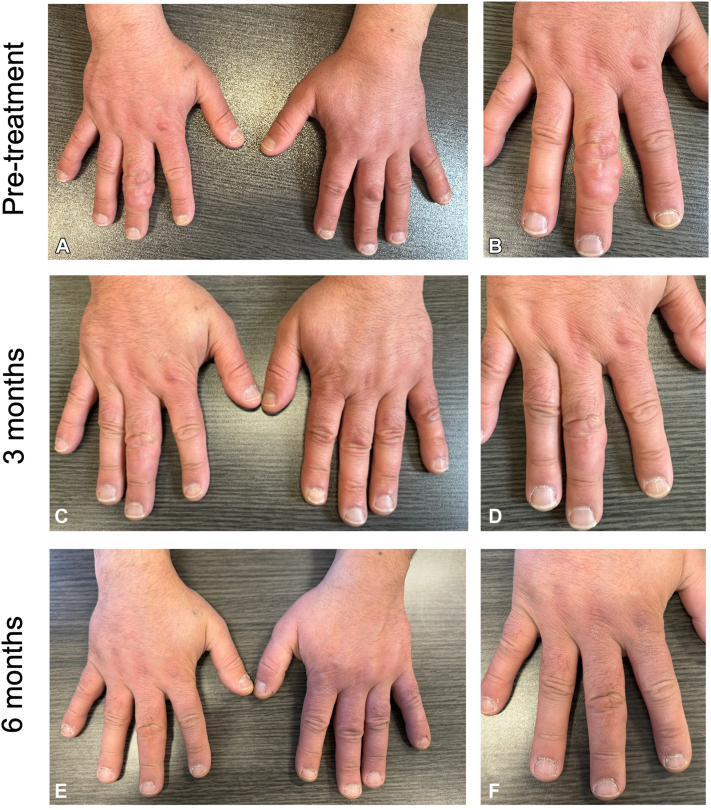
Clinical image of pseudorheumatoid nodules on the hands before treatment, at 3 months of treatment, and at 6 months of treatment. **A** and **B,** Multiple firm, skin-colored nodules are seen on the patient’s dorsal hands before treatment. **C** and **D,** Marked improvement of the nodules on the hands after 3 months of treatment with abrocitinib at 200 mg daily. **E** and **F,** Near complete resolution of the hand nodules after 6 months of treatment.

A 49-year-old male presented to our dermatology clinic with a 10-year history of painless nodules on the fingers, elbows, and heels. His medical history was notable for migraines treated with verapamil. Clinical examination revealed firm, skin-colored nodules on the dorsal fingers (Fig 1, *A* and *B*), elbows, and heels. Clustered nodules on the right third digit limited his range of motion in flexion.

**Fig 2:**
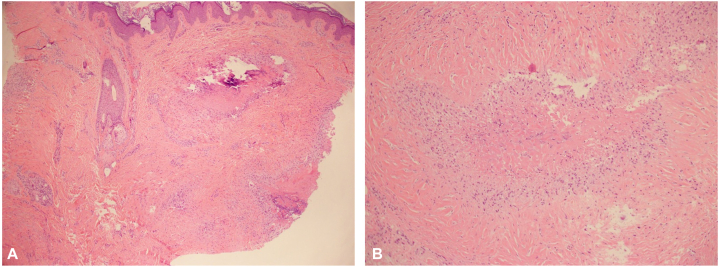
Histologic image of pseudorheumatoid nodules stained with hematoxylin-eosin. **A,** At low magnification (4×), palisading granulomas with central necrobiosis are found at all levels of the dermis and the subcutaneous tissue. **B,** At higher magnification (10×), a palisading necrobiotic granuloma surrounded by thickened collagen is observed.

A skin biopsy demonstrated multiple small dermal palisading granulomas with central fibrinoid necrosis, surrounded by prominent fibrosis (Fig 2, *A* and *B*). Alcian blue stain revealed no mucin. Tissue culture was negative, with no growth of bacterial, mycobacterial, or fungal organisms. Thorough evaluation by a rheumatologist showed no clinical or serological evidence of rheumatic disease. These findings were consistent with a diagnosis of pseudorheumatoid nodules.

Given the functional impairment caused by the lesions and the recent evidence of the role of the Janus kinase-signal transducer and activator of transcription (JAK-STAT) pathway in granulomatous disorders, the patient was started on abrocitinib, an oral JAK1 inhibitor, as a first-line therapy. Treatment was started at a daily dose of 200 mg. At the 3-month follow-up visit, there was marked improvement of the hand lesions (Fig 1, *C* and *D*) and complete resolution of the lesions on the elbows and heels. The patient regained full range of motion in his right third digit. The treatment was well tolerated with no adverse effects reported. At 6-month follow-up, near complete resolution of the hand lesions was observed (Fig 1, *E* and *F*), with no new lesions. The dose of abrocitinib was then reduced to 100 mg daily, with plans to pursue the treatment until complete resolution is maintained.

## Discussion

Pseudorheumatoid nodules are a variant of subcutaneous granuloma annulare with limited effective treatment options. Studies have demonstrated the upregulation of the JAK-STAT signaling pathway in the pathogenesis of granuloma annulare through the increase of inflammatory cytokines such as interferon-gamma and oncostatin M.^3^ The JAK-STAT pathway is emerging as an effective therapeutic target for various granulomatous disorders.^4^^,^^5^ Successful treatment of granuloma annulare has been reported with the JAK inhibitors tofacitinib,^3^ abrocitinib,^6^ and upadacitinib.^7^ However, there is a lack of long-term data on the maintenance of therapeutic response at withdrawal of the treatment. This case describes a rapid and effective response to abrocitinib, a selective oral JAK1 inhibitor, in the treatment of long-standing pseudorheumatoid nodules, a variant of subcutaneous granuloma annulare.

## Conclusion

This case further supports the role of JAK inhibitors as a promising therapeutic option for granulomatous skin diseases. Larger cohort studies or clinical trials with long-term follow-up are warranted to confirm these findings.

## Conflicts of interest

None disclosed.
